# Feasibility of using the Vero SBRT system for intracranial SRS

**DOI:** 10.1120/jacmp.v15i1.4437

**Published:** 2014-01-06

**Authors:** Manuela Burghelea, Dirk Verellen, Thierry Gevaert, Tom Depuydt, Kenneth Poels, Viorica Simon, Mark De Ridder

**Affiliations:** ^1^ Department of Radiotherapy UZ Brussel Brussels Belgium; ^2^ Faculty of Physics Babes‐Bolyai University Cluj‐Napoca Romania

**Keywords:** O‐ring gantry, treatment planning, intracranial radiosurgery

## Abstract

The Vero SBRT system was benchmarked in a planning study against the Novalis SRS system for quality of delivered dose distributions to intracranial lesions and assessing the Vero system's capacity for SRS. A total of 27 patients with one brain lesion treated on the Novalis system, with 3 mm leaf width MLC and C‐arm gantry, were replanned for Vero, with a 5 mm leaf width MLC mounted on an O‐ring gantry allowing rotations around both the horizontal and vertical axis. The Novalis dynamic conformal arc (DCA) planning included vertex arcs, using 90° couch rotation. These vertex arcs cannot be reproduced with Vero due to the mechanical limitations of the O‐ring gantry. Alternative class solutions were investigated for the Vero. Additionally, to distinguish between the effect of MLC leaf width and different beam arrangements on dose distributions, the Vero class solutions were also applied for Novalis. In addition, the added value of noncoplanar IMRT was investigated in this study. Quality of the achieved dose distributions was expressed in the conformity index (CI) and gradient index (GI), and compared using a paired Student's t‐test with statistical significance for p‐values ≤0.05. For lesions larger than 5 cm^3^, no statistical significant difference in conformity was observed between Vero and Novalis, but for smaller lesions, the dose distributions showed a significantly better conformity for the Novalis (ΔCI=13.74%, p=0.0002) mainly due to the smaller MLC leaf width. Using IMRT on Vero reduces this conformity difference to nonsignificant levels. The cutoff for achieving a GI around 3, characterizing a sharp dose falloff outside the target volume was 4 cm^3^ for Novalis and 7 cm^3^ for Vero using DCA technique. Using noncoplanar IMRT, this threshold was reduced to 3 cm^3^ for the Vero system. The smaller MLC and the presence of the vertex fields allow the Novalis system to better conform the dose around the lesion and to obtain steeper dose falloff outside the lesion. Comparable dosimetric characteristics can be achieved with Vero for lesions larger than 3 cm^3^ and using IMRT.

PACS number: 87.55.D

## I. INTRODUCTION

Stereotactic radiosurgery (SRS) is an essential primary treatment modality for cranial neoplasms. Many commercial radiosurgical systems are available, all relying on the same principle: achieve highly localized dose that conforms closely to the shape of the target, thus sparing a maximum amount of normal tissue. Recently, different frameless SRS systems based on image‐guided radiotherapy (IGRT) have been developed as an alternative to the invasive frame‐based immobilization, thus allowing for fractionated treatments with high‐precision positioning.^(1)^

Our institution introduced frame‐based linac SRS in 1992,^(2)^ and frameless image‐guided SRS using a robotic six degrees‐of‐freedom treatment couch with the Novalis SRS system (BrainLAB AG, Feldkirchen, Germany) in 2000.^(3)^ Three years ago, a new 4D IGRT system, specially designed for stereotactic body radiation therapy (SBRT) was installed. The Vero system, a joint product of MHI (Mitsubishi Heavy Industries Ltd., Tokyo, Japan) and BrainLAB (BrainLAB AG, Feldkirchen, Germany), utilizes a rotating, rigid ring structure for precise noncoplanar delivery and a gimbals linac capable of tumor tracking irradiation. The O‐ring structure incorporates different imaging and positioning systems that complete the design of the machine.

The Vero system's ability for radiosurgery is one unexplored potential of this novel system, and the current study aims to determine if it can be used as a backup system for Novalis in event of equipment failure. The high mechanical stability of the machine, the noncoplanar liberty offered by the O‐ring design, and the on‐board imaging capabilities made it suitable to be considered for intracranial stereotactic treatments. Until now, no other publication has investigated the Vero approaches for SRS using dynamic conformal arc (DCA) or noncoplanar intensity‐modulated radiation therapy (IMRT). The Vero SBRT system was benchmarked in a planning study against the Novalis SRS system for quality of radiosurgery dose distributions to intracranial lesions, to evaluate whether or not the Vero can be applied for SRS, and to identify the patients that might benefit from this approach.

## II. MATERIALS AND METHODS

### A. Treatment systems

The Novalis system is a 6 MV purpose‐built system for delivery of SRS, equipped with a micromultileaf collimator (mMLC) unit and in‐room image‐guided system.^(4)^ This mMLC unit consists of 26 leaf pairs of varying thickness with: 14 central leaf pairs of 3.0 mm wide each (projected at the isocenter plane), six intermediate pairs of 4.5 mm, and six peripheral pairs of 5.5 mm. The maximal field size at isocenter distance is $10\times 10\;{\text{cm}}^{2}$. The system is equipped with the ExacTrac X‐ray (BrainLAB AG, Feldkirchen, Germany) positioning system and accurate patient setup is provided by the six degrees robotic treatment couch.^(5)^

The Vero system consists of a 6 MV linear accelerator (linac) mounted on an O‐ring gantry that rotates around the patient by $\pm 185^{\circ }$ and, unlike C‐arm gantries, Vero can also rotate around the vertical axis $\left(\pm 60^{\circ }\right)$.^6^, ^7^ The latter allows noncoplanar delivery without couch rotations. The MLC is designed with 30 leaf pairs of 5.0 mm thickness with a field size of $15\times 15\;{\text{cm}}^{2}$. In addition, the linac and the 2D collimating system are mounted on the ring‐based gantry using a gimbals‐based mechanism that allows pan‐and‐tilt motion of the beam and submillimeter precision in dose delivery.^7^, ^8^ The system incorporates several imaging modalities in the O‐ring structure: EPID for MV portal imaging, and two orthogonal kV X‐ray tubes in combination with two flat panel detectors allowing patient imaging and positioning at any gantry and ring angle. The X‐ray system offers cone‐beam computed tomography (CBCT) and fluoroscopy, allowing real‐time imaging of moving targets. The Vero integrates IGRT and dynamic treatment delivery more efficiently in contrast to the Novalis. However, its design compromising on MLC leaf thickness (5.0 mm opposed to 3.0 mm close to the beam axis) and the clinical limitation of not being able to treat with vertex fields may affect dose distribution.

For this study, iPlan RT Dose (BrainLAB AG, Feldkirchen, Germany) version 4.5.1 was used as treatment planning platform for both Vero and Novalis systems. The pencil beam algorithm with a heterogeneity correction was used, and the dose was normalized to 100% at isocenter. The grid size of the dose‐volume histogram (DVH) calculation was set at $2\times 2\times 2\;{\text{mm}}^{3}$.

### B. Patient data

Patients that received intracranial SRS treatment for one lesion with the Novalis system were selected from the database for the period 2010‐2012. Magnetic resonance imaging (MRI) of 1 mm slice thickness and 2 mm computed tomography (CT) scans were obtained for all patients. Both MRI and CT scans were loaded into the treatment planning system and target delineation was performed by a physician. In addition to the reference Novalis plans and their analogous Vero comparative plans, a set of Vero plans was simulated using the Novalis MLC (Vero sim) to investigate in more detail the influence of MLC leaf width. To compensate for the lack of vertex fields and larger MLC leaf width, a set of step‐and‐shoot intensity‐modulated radiation therapy (IMRT)^(9)^ plans was added to the analysis.

The patient population presented relatively small brain lesions: six vestibular schwannomas, three meningiomas, one primary meningial melanoma, five arteriovenous malformations (AVMs), and 12 metastasis. The mean volume was 6.95 cm^3^, the median 3.92 cm^3^, ranging from 0.23 cm^3^ to 26.31 cm^3^. The average isocenter prescription dose was 22.3 Gy (ranging from 15 Gy to 37.5 Gy).

### C. Treatment planning characteristics

The main feature of DCA is that the MLC leaves move continuously, conforming to the beam's eye view projection of the target along the path of an arc. The noncoplanar arcs are defined by fixed couch rotations for the Novalis and fixed ring positions for the Vero whilst the gantry rotates around the patient.

**Figure 1:**
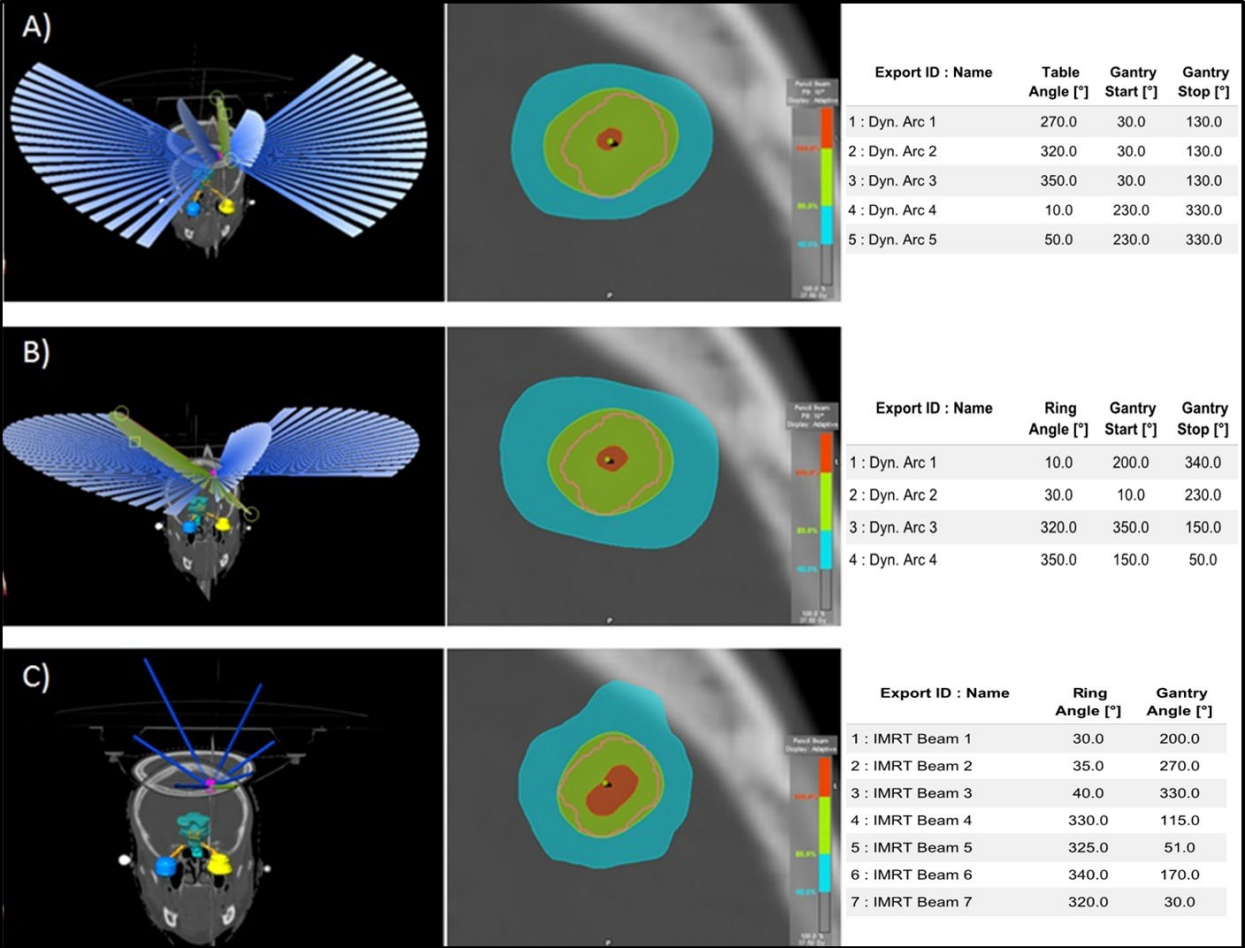
Illustration of the planning approaches, dose distribution and beam setup for the same patient: (a) Novalis template, (b) Vero “4 noncoplanar arcs approach”, and (c) Vero IMRT template.

The standard SRS treatment approach used in our department for the Novalis system consists of five noncoplanar arcs of 100° with a 40° couch (iso) rotation between the arcs. Sometimes, couch position or arc length are adapted to avoid traversing critical organs with a direct beam or to optimize dose gradients (Fig. 1(a)). The Novalis treatment planning usually includes vertex fields, using a 90° couch rotation. For the Vero system, due to the mechanical limitations of the O‐ring gantry, the vertex fields cannot be reproduced; therefore, alternative noncoplanar solutions were investigated to treat cranial lesions. The number of arcs was variable, depending on the target volume, shape, and localization. Initially the five‐arc technique was also applied for the Vero, but gradually alternative treatment approaches were explored minimizing the number of arcs and enlarging the arc's length (Fig. 1(b)).

As literature demonstrated the advantage of smaller leaf width,^10^, ^11^ this study also compared the 3 mm vs. 5 mm MLC for all the Vero plans by simulating them on the Novalis, keeping all the arc parameters identical and compensating the ring rotation with the couch rotation. Possible couch collisions were neglected for this simulation. In all cases, the leaf edges were manually adapted to make sure that the target volume was completely encompassed by the prescription isodose surface.

For the Vero IMRT plans, a step‐and‐shoot approach was applied.^(9)^ For this analysis, a template of seven noncoplanar beams (respecting the O‐ring limitations) was created and used for all patients (Fig. 1(c)).

### D. Plan comparison

Treatment planning intercomparisons were performed using the following dose‐volume histogram (DVH) parameters: conformity index, homogeneity index, and gradient index.

Many conformity indices are reported in literature^(12)^ and for this paper, the conformity index (CI) suggested by Paddick^(13)^ was used as it simultaneously takes into account dose coverage of the target volume and irradiated volume of healthy tissue:

(1) $$\text{CI}=\frac{{\text{TV}}_{\text{PIV}}}{\text{TV}}\times \frac{{\text{TV}}_{\text{PIV}}}{\text{PIV}}$$

where $TV_{PIV}$ is the target volume (TV) within the prescribed isodose volume (PIV). The first fraction defines the coverage quality of the target. The second fraction defines the volume of healthy tissue receiving a dose larger or equal with the prescribed dose. In the best scenario the CI would be equal to 1.

For the dose distribution inside the lesion, the following homogeneity index (HI)^(14)^ was used:

(2) $$\text{HI}=\frac{D_{2}-D_{98}}{D_{P}}$$

where $D_{p}$ is the prescription dose, and $D_{2}$ and $D_{98}$ represent the doses to 2% and 98% of the PTV, respectively. $D_{98}$ indicates that at least 98% of the target volume receives this dose; hence $D_{2}$ and $D_{98}$ are considered to represent the maximum and minimum doses. Lower HI values indicate a more homogeneous target dose.

The gradient index (GI) is introduced to measure the dose falloff outside the target and to determine the optimal treatment plan obtaining the steepest dose falloff for any given isodose configuration:^(15)^

(3) $$\text{GI}=\frac{V_{40}}{V_{80}}$$

The proposed GI is the ratio of the volume of half the prescription isodose to the volume of the prescription isodose, so a low GI would indicate a sharp dose falloff. For a plan normalized to the 80% isodose line, it is the ratio of the 40% isodose volume $\left(V_{40}\right)$ to the 80% isodose volume $\left(V_{80}\right)$.

All treatment plans were optimized to ensure clinically acceptable doses to organs at risk (such as brainstem, eyes, and optic nerves). To better analyze the difference between the diverse techniques, the patients were categorized in two groups according to the target volume: smaller and larger than 5 cm^3^. A normal distribution for all data was assumed, so the analysis was performed using a paired Student's *t*‐test to determine if there was a significant difference in any of the parameters analyzed. The difference was considered statistically significant for p‐values ≤0.05.

## III. RESULTS

### A. conformity index

**Figure 2:**
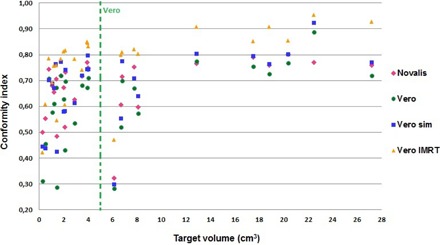
Conformity index as a function of target volume for the different approaches. The dashed green line represents the CI threshold between Vero and Novalis. Novalis: 5 DCAs of $\pm 100^{\circ }$ length with $\pm 40^{\circ }$ couch rotation between the arcs (includes a vertex arc with $\pm 90^{\circ }$ couch rotation); Vero: 4‐5 DCAs of $\pm 120^{\circ }$ length with $\pm 30^{\circ }$ ring rotation between the arcs (no vertex arc); Vero sim: identical arcs arrangement as Vero, with 3 mm MLC; Vero IMRT: 7 “step‐and‐shoot” IMRT beams in a noncoplanar template using $\pm 30^{\circ }$ ring rotation.

The different conformity values obtained with the investigated approaches are presented in Fig. 2. The CI for Novalis ranged between 0.32 and 0.80, with a mean of 0.67±0.11. Vero conformity indices varied from 0.28 to 0.89, with a mean of 0.62±0.16. Using the Novalis MLC on the Vero system, an increase of CI to 0.68±0.14 (0.30 to 0.92) was observed. A mean CI of 0.77±0.13 (0.42 to 0.95) for Vero IMRT method was acquired.

The mean difference with the standard deviation, mean percent difference, and p‐value are presented in Table 1. There was a statistical significant difference in the CI (p=0.0002) for the Vero vs. Novalis for lesions smaller than 5 cm^3^, while for lesions larger than 5 cm^3^ the difference was not significant (p=0.17). In the case of Vero simulated with Novalis MLC (Vero sim) vs. Novalis, no statistical difference in conformity was obtained. When the inverse planning Vero IMRT approach was used, an improved CI was achieved in all cases compared to Novalis, with a mean per cent difference of 0.05%.

The results indicate a tendency between target volume and CI for the different approaches, the difference decreasing from small lesions to larger lesions (Table 1). For example, in the MLC‐based comparison (Vero vs. Vero sim), the mean CI percent difference decreased from 11. 44% to 6.10%.

**Table 1:** Difference in CI as a function of target volume for the analyzed approaches. Differences are presented as mean CI difference±standard deviation, mean percent difference, and p‐value

*Target Volume*	*Vero vs. Novalis*	*Conformity Index Vero sim vs. Novalis*	*Vero vs. Vero sim*	*Vero IMRT vs. Novalis*
$<5{\text{cm}}^{3}$	−0.07±0.06	−0.1±0.05	−0.06±0.06	0.07±0.05
	−13.74%; p=0.0002	−2.36%; p=0.48	−11.44%; p=0.0006	0.05%; p=0.0002
$>5{\text{cm}}^{3}$	−0.02±0.05	0.02±0.06	−0.04±0.02	0.13±0.05
	−4.57%; p=0.17	1.52%; p=0.36	−6.10%; p=0.00001	0.10%; p=0.00001

### B. Homogeneity index

For the dose variation inside the lesion (HI), the Vero and Novalis techniques proved to be comparable. Both systems presented a mean value of 0.22±0.03; the Vero values ranged from 0.19 to 0.29 and the Novalis from 0.18 to 0.32. No statistical significant difference was observed. The HI values for the Vero sim plans ranged from 0.18 to 0.33, with a mean value of 0.24±0.04.

### C. Gradient index

**Figure 3:**
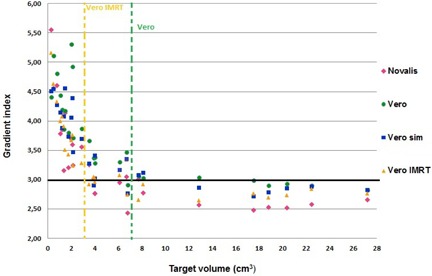
Gradient index as a function of target volume for the different approaches. The green and orange dashed lines represent the Vero DCA and Vero IMRT thresholds for GI>3. Novalis: 5 DCAs of $\pm 100^{\circ }$ length with $\pm 40^{\circ }$ couch rotation between the arcs (includes a vertex arc with $\pm 90^{\circ }$ couch rotation); Vero: 4‐5 DCAs of $\pm 120^{\circ }$ length with $\pm 30^{\circ }$ ring rotation between the arcs (no vertex arc); Vero sim: identical arcs arrangement as Vero, with 3 mm MLC; Vero IMRT: 7 “step‐and‐shoot” IMRT beams in a noncoplanar template using $\pm 30^{\circ }$ ring rotation.

The Novalis plans were consistently superior to those of Vero and Vero sim regarding normal tissue sparing (Fig. 3). The mean values were 3.27±0.77 (2.43 to 5.56), 3.70±0.74 (2.82 to 5.11), and 3.5±0.63 (2.71 to 4.56) for Novalis, Vero, and Vero sim, respectively. For the Vero vs. Novalis comparison, a statistically significant difference was observed for small (12.78%) and large lesions (12.29%) alike. This difference decreased somewhat to 6.09% and 9.31% by using the 3 mm MLC for the Vero (Vero sim vs. Novalis), but was still significant (Table 2). For noncoplanar IMRT (3.34±0.66), similar results were obtained as Novalis, with no significant difference for lesions larger than 3 cm^3^.

From Paddick's review,^(15)^ a GI of approximately 3 reflects a favorable steep dose gradient for SRS. In our study, this threshold was observed for lesions larger than 4 cm^3^ and 7 cm^3^ for Novalis and Vero, respectively. The same threshold was observed for Vero simulated cases ($>7\;{\text{cm}}^{3},\;GI∼3$). Appling IMRT, the Vero was able to better limit the dose outside the lesion presenting comparable results with the Novalis approach (Fig. 3).

**Table 2:** Difference in GI as a function of target volume for the analyzed approaches. Differences are presented as mean GI difference±standard deviation, mean percent difference, and p‐value

*Target Volume*	*Vero vs. Novalis*	*Gradient Index Vero sim vs. Novalis*	*Vero vs. Vero sim*	*Vero IMRT vs. Novalis*
$<5{\text{cm}}^{3}$	0.47±0.58	0.2±0.47	0.28±0.38	0.01±0.33
	12.78%; p=0.01	6.09%; p=0.05	6.72%; p=0.01	0.02%; p=0.88
$>5{\text{cm}}^{3}$	0.35±0.14	0.26±0.08	0.09±0.10	0.15±0.18
	12.29%; p=0.00001	9.31%; p=0.000001	2.98%; p=0.02	0.41%; p=0.02

**Figure 4:**
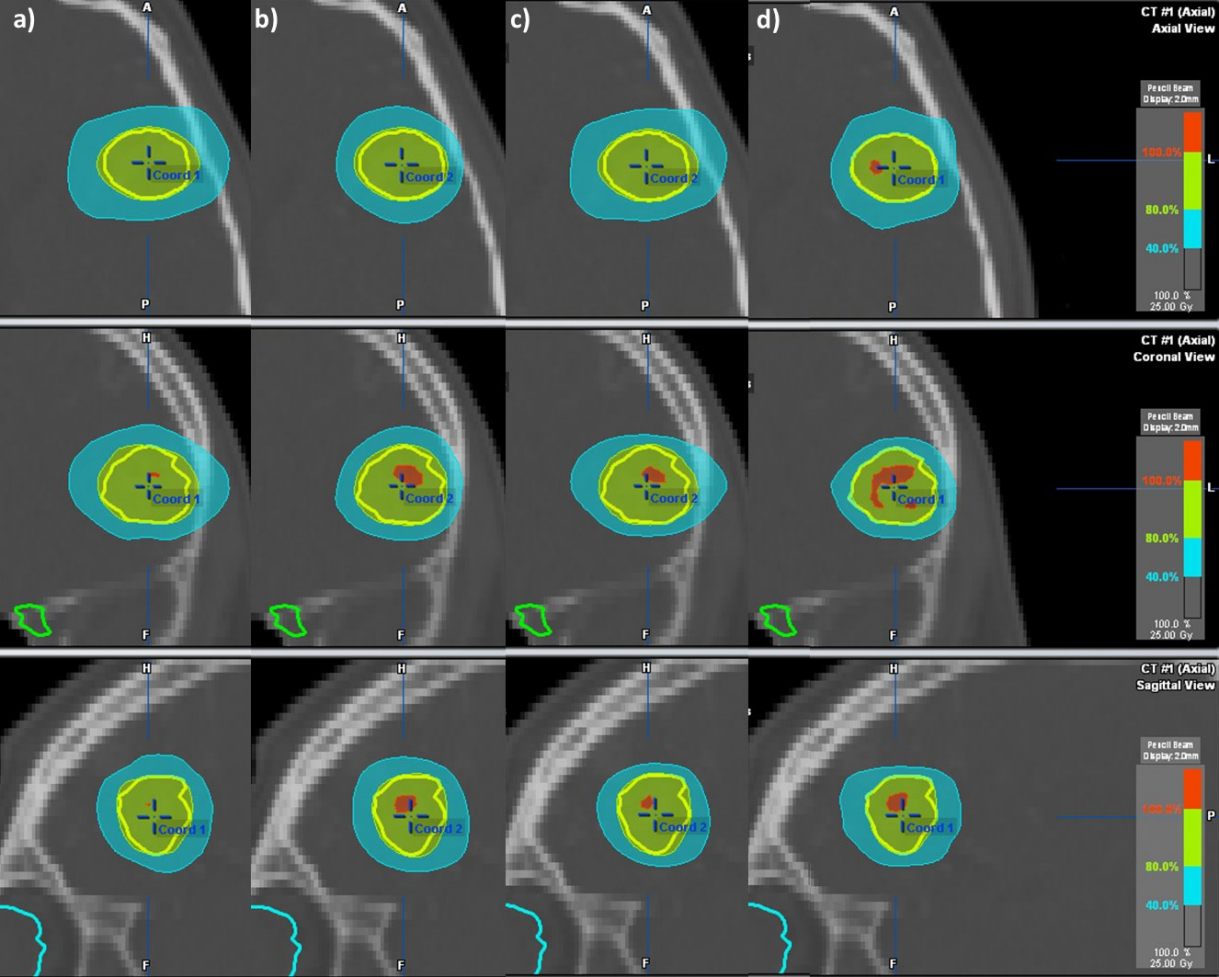
Example of the dose distribution obtained for a typical patient with a 4.05 cm^3^ lesion using: (a) Vero, (b) Novalis, (c) Vero sim, and (d) Vero IMRT.

Dose distributions obtained for a typical patient using the Vero (without the vertex fields), Novalis, Vero sim, and Vero IMRT approaches are presented in Fig. 4. In the Vero case, an ellipsoidal‐shaped dose distribution around the lesion could be observed in the axial plane. The presence of the vertex fields generated a more spherical distribution, and the Novalis approach yielded a better gradient compared to Vero. The use of 3 mm MLC compared to 5 mm MLC added dosimetric advantage in conformity and normal tissue sparing for Vero sim, but the dose shape remained characteristic to the vertex field's absence. For Vero IMRT, where multiple noncoplanar beams and inverse planning were used to sculpt the dose around the lesion, a dosimetric improvement could be observed.

## IV. DISCUSSION

The dosimetric differences between Novalis and Vero plans for a broad range of brain tumors were analyzed in an effort to determine whether the Vero system is suitable for intracranial stereotactic radiosurgery. In contrast with the “standard Novalis approach”, different approaches to treat cranial cases were investigated in the planning methodology for Vero: first to compensate for the absence of the vertex fields and smaller MLC leaf width and, secondly, to explore different gantry‐ring combinations to realize acceptable dose distributions characteristic for a radiosurgery technique.

In general, better conformity was obtained with the Novalis system compared to the Vero system as was to be expected. For lesions smaller than 5 cm^3^, the Novalis CI presented a statistically significant superiority, but for targets larger than 5 cm^3^ no statistically significant difference was observed. MLC leaf width was identified to be an important factor, as demonstrated by the Vero plans simulated on the Novalis where the 3 mm MLC yielded significantly higher CI values compared to 5 mm MLC. Several studies on the dosimetric impact of MLC leaf widths are reported in literature^16^, ^17^ confirming dosimetric advantage with smaller leaves for different tumor shapes, supporting our analysis. Furthermore, a decrease in the difference in conformity with increasing target volume was observed in our study, again confirming previous reports.^10^, ^11^, ^18^

Both systems investigated in this report presented comparable values for the dose distribution inside the lesion (with a mean HI of 0.22). Dose homogeneity is a rather controversial issue in radiosurgery and reflects more or less a department's treatment strategy. In this study, dose homogeneity was considered as a separate treatment parameter, and for each individual plan the isocenter dose was adjusted to reach treatment constraints.

Using DCA, the Novalis and Vero systems were able to obtain an adequate GI for lesions lager than 4 cm^3^ and 7 cm^3^, respectively. The GI for Vero improved by using 3 mm MLC leafs, but not significantly, indicating that the main source of difference in GI between the Novalis and the Vero system could be attributed to the presence or absence of the vertex arc.

To reduce the 7 cm^3^ threshold for obtaining an acceptable GI level, noncoplanar IMRT approach was introduced. An improvement in GI was observed for all cases compared to Vero DCA. When these results were compared to Novalis, comparable GI values were obtained for small lesions.

An alternative solution that could be evaluated in future studies is to investigate nonconventional patient setups. Instead of the usual supine position for cranial treatments, the patient might be placed sideways, offering more degrees of freedom for the noncoplanar delivery with the O‐ring rotation.

Both systems presented differences in leaf leakage (Vero 0.13%, Novalis 1.4%)^(8)^ and the quality index of the beam (Vero 0.67, Novalis 0.65), which was considered to be less relevant for this planning study. The difference in leaf width, the planning setup, and the vertex arc were the major parameters considered to influence the conformity and dose gradient.

## V. CONCLUSIONS

This study investigated whether the Vero system can achieve dose distributions characteristic for stereotactic radiosurgery with a sharp dose falloff outside the target and tight conformity around the lesion comparable to those obtained by the Novalis system.

The analysis demonstrated that the Novalis SRS system presents a dosimetric advantage over the Vero SBRT system for lesions smaller than 7 cm^3^ with respect to treatment conformity and normal tissue sparing. For lesions larger than 7 cm^3^, the Vero system was able to achieve the required dosimetric characteristics for stereotactic radiosurgery (CI comparable with Novalis, GI∼3). Furthermore, using noncoplanar IMRT, this threshold was reduced to 3 cm^3^. The results confirmed the importance of leaf width for conformity, and the influence of vertex fields in dose gradient. A Vero system with thinner MLC leafs would improve the dose conformity (CI); however, GI would still be inferior to a Novalis system due to the absence of the vertex fields if DCA were to be used.

## ACKNOWLEDGMENTS

This collaborative work was supported by the Flemish government through the Hercules foundation, the “Fonds voor Wetenschappelijk Onderzoek ‐ Vlaanderen” Grants G.0486.06 and G.0412.08, and corporate funding from BrainLab AG.

## Supporting information

Supplementary Material

Supplementary Material

Supplementary Material
