# Supplementary figures and images for: Feasibility of using the Vero SBRT system for intracranial SRS
Source: J Appl Clin Med Phys. 2014 Jan 6;15(1):90–9. doi: 10.1120/jacmp.v15i1.4437 (PMC5711224; doi:10.1120/jacmp.v15i1.4437)

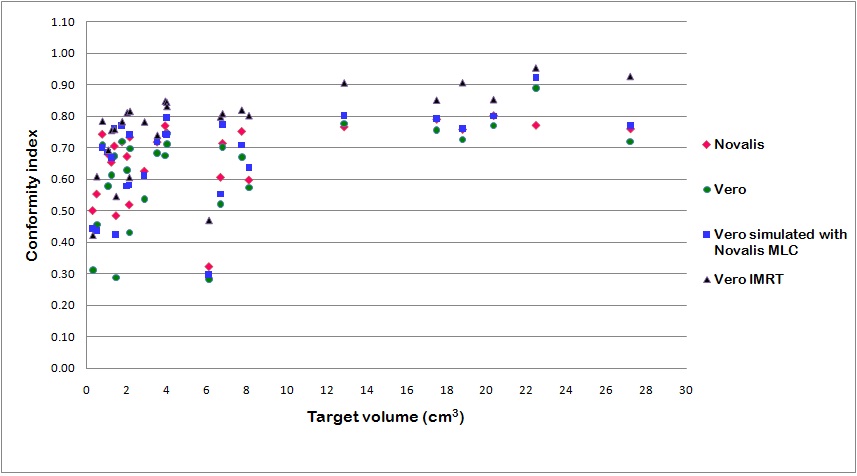

Supplement: Supplementary file 1 — Supplementary Material [file ACM2-15-090-s001.jpg]

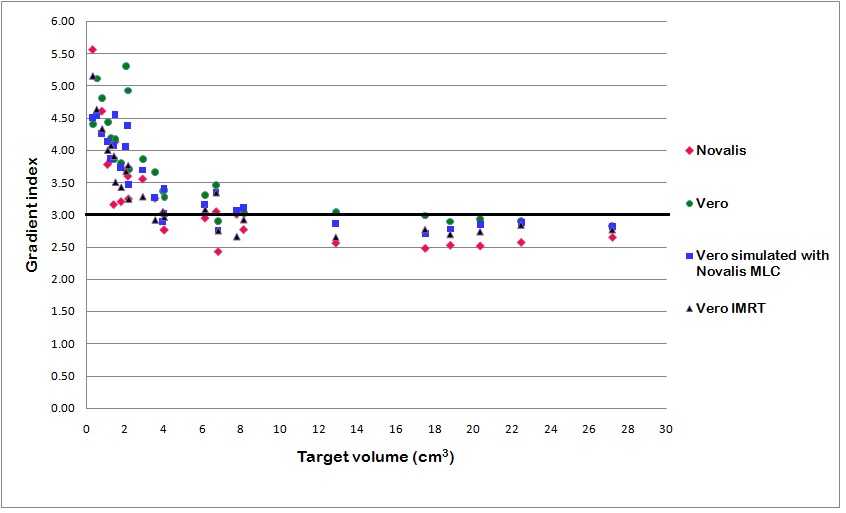

Supplement: Supplementary file 2 — Supplementary Material [file ACM2-15-090-s002.jpg]

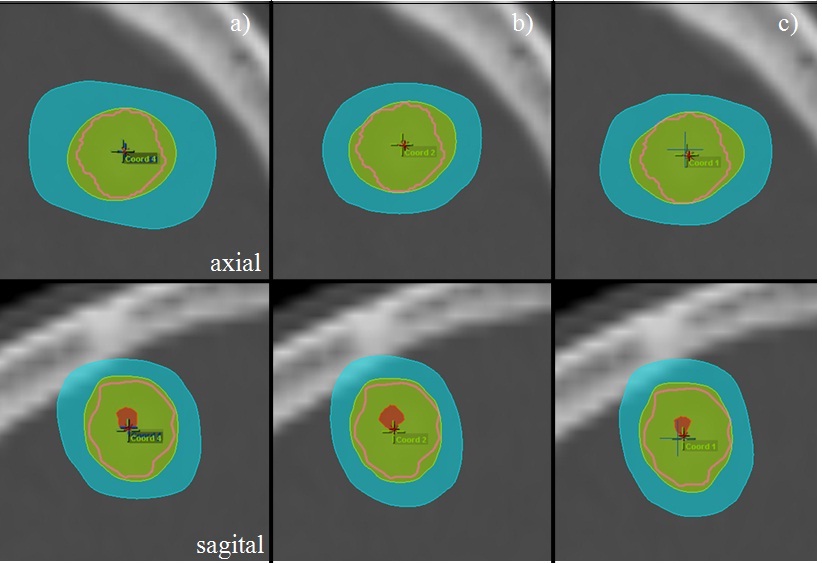

Supplement: Supplementary file 3 — Supplementary Material [file ACM2-15-090-s003.jpg]
